# A probabilistic method for identifying rare variants underlying complex traits

**DOI:** 10.1186/1471-2164-14-S1-S11

**Published:** 2013-01-21

**Authors:** Jiayin Wang, Zhongmeng Zhao, Zhi Cao, Aiyuan Yang, Jin Zhang

**Affiliations:** 1 Department of Computer Science and Technology, Xi'an Jiaotong University, Xi'an, P.R.China; 2 Computer Science and Engineering Department, University of Connecticut, Storrs, Connecticut 06269-2155, USA

## Abstract

**Background:**

Identifying the genetic variants that contribute to disease susceptibilities is important both for developing methodologies and for studying complex diseases in molecular biology. It has been demonstrated that the spectrum of minor allelic frequencies (MAFs) of risk genetic variants ranges from common to rare. Although association studies are shifting to incorporate rare variants (RVs) affecting complex traits, existing approaches do not show a high degree of success, and more efforts should be considered.

**Results:**

In this article, we focus on detecting associations between multiple rare variants and traits. Similar to *RareCover*, a widely used approach, we assume that variants located close to each other tend to have similar impacts on traits. Therefore, we introduce elevated regions and background regions, where the elevated regions are considered to have a higher chance of harboring causal variants. We propose a hidden Markov random field (HMRF) model to select a set of rare variants that potentially underlie the phenotype, and then, a statistical test is applied. Thus, the association analysis can be achieved without pre-selection by experts. In our model, each variant has two hidden states that represent the causal/non-causal status and the region status. In addition, two Bayesian processes are used to compare and estimate the genotype, phenotype and model parameters. We compare our approach to the three current methods using different types of datasets, and though these are simulation experiments, our approach has higher statistical power than the other methods. The software package, *RareProb *and the simulation datasets are available at: http://www.engr.uconn.edu/~jiw09003.

## Introduction

In most existing genetic variant association studies, "common trait, common variants", which asserts that common genetic variants contribute to most of traits (disease susceptibilities), serves as the central assumption. Researchers have successfully identified some significant associations between common single nucleotide polymorphisms (SNPs) and disease traits [1]. However, despite the enormous efforts expended on association studies of complex traits, common genetic variants only show a moderate influence on different phenotypes in many reported disease associations and consequently have limited diagnostic value [2,3]. While the identification of common variants creates a dilemma, known as "common trait, rare variants", an alternative hypothesis, which asserts that multiple rare variants with moderate to high penetrances may collectively influence disease susceptibilities, has been suggested in some literatures [3-5]. Rare variants are defined as those whose minor allele frequencies (MAF) are less than or equal to 0.01 (≤ 10^-2^). Although some rare variants associated with Mendelian diseases have been identified, more often, the allelic population attributable risk (PAR), which describes a small reduction in the incidence that would be observed in unexposed samples compared to the actual exposure pattern, is low. The odds ratio (OR), a measure of the strength of association or non-independence between two binary data values, is also low. Moreover, based on the "common trait, rare variants" hypothesis, in many cases, a set of rare variants, instead of just one variant, should be identified to fully explain the genetic influence. Both the single-variant test [6] and the multiple-variant test [7] have been applied to rare variant association studies. However, due to the reasons outlined above, neither of them shows satisfactory power in obtaining associations. Although more and more attention is being focused upon rare variants, there has only been limited success thus far [8-10].

Alternatively, the collapsing strategy, also called the "burden-based test", is another approach for rare variants association studies. Most of the collapse-based approaches build on the "recessive-set" genetic model, in which the predisposing haplotype contains mutation(s) in at least one variant [11]. Multiple rare variants in the same locus are collapsed, based on different standards, then statistical tests are applied. The locus here is defined as a selected region that consists of a group of candidate rare variants [9,12-14]. However, it is argued that existing collapse-based approaches assume all rare variants implicitly influencing the phenotype in the same direction and with the same magnitude [10,15]. Researchers have observed that any given rare variant could have no effect, could be causal, or could be protective for the endpoints (traits) [15]. For example, some low-frequency variants in African Americans *PCSK9 *can have a substantial effect on serum *Low-Density Lipoprotein Cholesterol *(LDL-C) by increasing the risk of or protecting against myocardial infarction [16-18].

Collapse-based approaches have low statistical powers when "causal", "neutral" and "protective" variants are combined [13,15,19]. To overcome this weakness, some approaches [9,14] assume that the rare variants are well selected by experts, while weighting of each variant is another widely used strategy [9,11,14]. In a recent study, Bhatia and others [19] suggest the development of a "model-free" approach, *RareCover*, that only collapses a subset of potentially causal variants from all of the given variants. Here, the "model" refers to the genetic association model that consists of the pre-selection candidate variants.

Motivated by *RareCover*, in this article, we focus on rare variant association analysis without any pre-selection of candidate variants. We propose a probabilistic approach, *RareProb*, to make selections using a Markov random field (MRF) model and identify multiple causal rare variants that influence a dichotomous phenotype using statistical tests. Our approach considers both the causal and the protective variants, which distinguishes it from the previous study *RareCover*, and it is therefore a robust predictor of the direction and the magnitude of the genetic effects. Moreover, inspired by the weight-sum approaches [9,11,14], we also weight each variant; however, we not only consider the likelihood of a variant being causal but also compute the pair-wise likelihood of candidate variants being collapsed together. Note that although it is difficult to observe, relatively low interactions (e.g., linkage disequilibrium) are expected between rare variants [4,11,13,20]. Furthermore, in regression-based association methods, genetic similarities are often used to reduce the dimensions of the regression models. Therefore, we introduced two kinds of genetic regions, the elevated region and the background region, in our model analysis; the elevated region has a higher probability of harboring a causal variant. This assumption that the causal variants are often located close to each other is often used, e.g. slide windows in *RareCover *[19]. However, the regions are more flexible than a preset slide window, as in *RareCover*.

We adopt the "dominant" and "recessive set" genetic model, which are also used in [9-11,14,15,19]. In the dominant and recessive-set model, the predisposing genotype harbors the mutation(s) in at least one variant on any of the two haplotypes. Therefore, for one genotype, there are two possible allelic values at each variant: one denotes that both haplotypes carry a wide-type allele, while the other denotes that at least one haplotype carries a mutant. In our method, each variant has two hidden states, causal/non-causal status and elevated/background region status. The MRF includes the hidden states, emission probabilities and transition probabilities. The emission probabilities bridge the hidden states and the genotypes, while the transition probabilities link the two hidden states. Following the pseudo-likelihood estimation method [21], we infer the model parameters and all of the hidden states. The simulation experiments show that our approach outperforms *RareCover, RWAS *[14] and *LRT *[9] on different parametric settings. In particular, *RareProb *obtains better results on large-scale data.

## Methods

### Notions and model overview

Suppose we are given *M *rare variants (allelic sites) on a set of *N *genotypes. Let *s_i _*denote the allelic value of the site *s *on the genotype *i *(1 ≤ *i *≤ *N*, 1 ≤ *s *≤ *M*), where *s_i _*= 0 means both haplotypes of *i *have the wild type allele, while *s_i _*= 1 means at least one haplotype has a mutant allele. Each genotype carries a dichotomous phenotype. Let vector *P *denotes the phenotypes, where *P_i _*= 1 represents that *i *is affected by the phenotype trait (being a case), while *P_i _*= 0 represents that *i *is a control.

The core of our approach is a Markov random field (MRF) model. We first introduce four key components of modeling this MRF:

• The observed data of this MRF consist of all of the genotypes and phenotypes.

• There are two unknown states for each site: one is the causal or non-causal status and the other is the region location status. Here, we define them as the hidden states of this Markov random field. Let a latent vector *R *represent the region status, where *R_s _*= 1 denotes that the site *s *is located in an elevated region, while *R_s _*= 0 denotes the *s *is located in a background region. Additionally, let a latent vector *X *represent the causal/non-causal status, where *X_s _*= 1 if the site *s *is causal (contributes to the phenotype); otherwise, *X_s _*= 0. Probabilistic functions are designed to present the probabilities of each hidden state. The *RareProb *framework is able to incorporate prior information, obtained by different software tools, e.g. *Align-GVGD *[22] and *SIFT *[23], etc, by updating initial *X *vector and *R *vector.

• A neighborhood system is required in the MRF model to describe the interactions among hidden states. Details of the hidden states and neighborhood system are shown in the section "Estimation of the hidden states in HMRF".

• There are two kinds of probabilities in the MRF model: emission probabilities and transition probabilities. Emission probabilities bridge the relationships among genotypes, phenotypes and hidden states. Moreover, hidden states *X *and *R *are not independent of each other, as the relationships between the hidden states are described by the transition probabilities. The conditional probability *P*(*X_s _*= 1|*R_s _*= 1) denotes the probability that the site *s *is a causal site when it is located in an elevated region, while *P*(*X_s _*= 0|*R_s _*= 1) denotes the probability that the site *s *is non-causal when it is located in an elevated region. Similarly, another two conditional probabilities, *P*(*X_s _*= 1|*R_s _*= 0) and *P*(*X_s _*= 0|*R_s _*= 0), present the probabilities of being causal or non-causal if the site is located in a background region. Details of the emission probabilities are shown in the section "Estimation of the emission probabilities in HMRF", and the transition probabilities are shown in the section "Estimation of the transition probabilities in HMRF".

The central thesis of our approach is that causal rare variants, which should be collapsed together, are treated as one random vector variable with certain dimensions. Then, the probability of this bunch of causal rare variants becomes the probability of one variable being associated with the phenotype. Based on the Markov-Gibbs equivalence [21], the probability of this random variable can be decomposed into the sum of *clique potentials*. The *first-order clique potentials *describe the probability of one variant being causal, while the *second-order clique potentials *measure the pair-wise genetic similarities, which share the idea of the kernel machine in regression frameworks [10,24,25]. The neighborhood system in the MRF model consists of clique potentials. In our approach, we select that the neighborhood system only contains the first-order and the second-order clique potentials because there is scanty evidence supporting the biological or medical scenario of high-order potentials. For each variable, the MAFs and model parameters can be estimated by maximizing the likelihoods of the genotypes. Then, the probability of the variable and the variable itself can be updated by MAFs and model parameters. Two or three iterations can be applied if needed for the convergence of the MRF. Thus, our approach selects a subset of candidate causal variants by updating the variables and avoids the weakness of the same magnitude effect assumption because the neighborhood system is able to describe both the "causal" and "protective" variants.

### Estimation of the hidden states in HMRF

#### Neighborhood system

Assume there are *N/*2 cases and *N/*2 controls among all of the genotypes (if the number of cases is not equal to the number of controls, then all of the results still can be used by applying noncentrality parameters). At a certain variant *s*, let *θ_s _*denote the MAF for the cases, and let the number of genotypes in cases that carry at least one mutant allele be $c_{s}^{+}$. Let *ρ_s _*denote the MAF for the controls, and let the number of genotypes in controls that carry at least one mutant allele be $c_{s}^{-}$. Then, we can draw two binomial distributions for the cases and the controls [9,14]: $c_{s}^{+}\sim \text{Bin}\left(\frac{N}{2}\theta_{s}\right)$ and $c_{s}^{-}\sim \text{Bin}\left(\frac{N}{2},\rho_{s}\right)$, where $f\left(c_{s}^{+}|\theta_{s}\right)=C_{\frac{N}{2}}^{c_{s}^{+}}\theta_{s}^{c_{s}^{+}}{\left(1-\theta_{s}\right)}^{\frac{N}{2}-c_{s}^{+}}$ and $f\left(c_{s}^{-}|\rho_{s}\right)=C_{\frac{N}{2}}^{c_{s}^{-}}\rho_{s}^{c_{\text{s}}^{-}}{\left(1-\rho_{S}\right)}^{\frac{N}{2}-c_{s}^{-}}$. Thus, for a site *s*, the statistic of the difference between *θ *and *ρ *is

$$z_{s}=\frac{2\left({\hat{\theta }}_{s}-{\hat{\rho }}_{s}\right)}{\sqrt{\frac{2}{N}}\sqrt{\left({\hat{\theta }}_{s}+{\hat{\rho }}_{s}\right)\left(2-{\hat{\theta }}_{s}-{\hat{\rho }}_{s}\right)}}$$

where ${\hat{\theta }}_{s}=\frac{c_{\text{s}}^{+}}{N/2}$ is the estimation of *θ_s _*and ${\hat{\rho }}_{s}=\frac{c_{\text{s}}^{-}}{N/2}$ is the estimation of *ρ_s_*. Similar to the linear kernel function, which calculates genetic similarities [10], we measure the likelihood between pairwise rare variants, which denotes how likely two variants would be collapsed together. For two variants *s *and *s'*, we define *ω_s,s' _*as the likelihood of collapsing as follows:

$$\omega_{s,s^{\prime }}=\frac{2z_{s}z_{s^{\prime }}}{z_{s}^{2}+z_{s^{\prime }}^{2}}$$

The *ω *function has the following properties: (1) When both *s *and *s' *are causal variants, due to the PAR, *ω_s,s' _*locates in the interval (0, 1]. (2) If one variant is "causal" but the other is "protective", the likelihood takes on a negative value. (3) The likelihood encourages the collapse of the variants with similar PAR. Those rare variants whose MAFs increase rapidly in some cases, as we mentioned before, could be identified by single-site tests or pair-wise tests, which are often not considered in collapsing models [8]. Let *ω*.,. be the weight of two neighbors. The closer the statistics *z_s _*and *z_s' _*are, the larger the likelihood will be. And thus, the neighborhood system is built up.

#### Hidden states

Rare variant *s *is either located in an elevated region or in a background region. Thus, we define the probability (Bayesian classifier) of *s *as

$$p\left(X_{s}|X_{n\left(s\right)}\right)\propto \text{exp}\left(\gamma p\left(X_{s}|R_{s}\right)X_{s}+\eta \underset{s^{\prime }\in n\left(s\right)}{\sum }\omega_{s,s^{\prime }}p\left(X_{s^{\prime }}|R_{s^{\prime }}\right)X_{s^{\prime }}\right)$$

where *n *(*s*) denotes the neighbors of *s*. *γ *and *η *are two MRF parameters. *γ *represents how strongly the status of *X_s _*affects the probability of *X_s_*, while *η *represents how strongly the neighbors of *s *affect the probability of *X_s_*. Here, we limit *η >*0, which encourages the pair-wise weights and prevents them from counteracting the negative weights. Thus, the joint probability of the latent vector *X *is $p\left(X;\Phi \right)\propto \text{exp}\left(\gamma \sum_{s}^{M}p\left(X_{s}|R_{s}\right)+\eta \sum_{s^{\prime }\in n\left(s\right)}\omega_{s,s^{\prime }}p\left(X_{s^{\prime }}|R_{s^{\prime }}\right)\right)$, where Φ = (*γ, η*). As the variants in different subsets (different collapsing groups) are conditional independent, this joint probability covers all of the probabilities of the random variables (collapsing groups). Similarly, the probability of *s *located in an elevated region can be represented by

$$p\left(R_{s}|R_{n\left(s\right)}\right)\propto \text{exp}\left(\tau R+\upsilon \underset{s^{\prime }\in n\left(s\right)}{\sum }\omega_{s,s^{\prime }}R_{s^{\prime }}\right)$$

and the joint probability of latent vector *R *can be represented by $p\left(R;\Phi_{R}\right)\propto \text{exp}\left(\tau \sum_{s}^{M}R+\upsilon \sum_{s,s^{\prime }}\omega_{s,s^{\prime }}R_{s^{\prime }}\right)$, where Φ*_R _*= (*τ, υ*)·*τ *and *υ *are two MRF parameters. We also limit *υ >*0, which encourages the pair-wise weights and prevents them from counteracting the negative weights.

### Estimation of the emission probabilities in HMRF

We now estimate the emission probabilities to relate *X *and *R *with the observed data. As linkage disequilibrium is rarely observed between rare variants [8], the vector consists of the allelic values from one variant that is *conditionally *independent from the others, when a particular *X *is given. Thus, the joint conditional probability of all of the genotypes is

$$p\left(Y|X\right)=\text{exp}\left(\overset{M}{\underset{s=1}{\sum }}p\left(Y_{s}|X_{s}\right)\right)$$

If *X_s _*= 1, due to the PAR, *θ_s _*≠ *ρ_s_*. We place a prior distribution on *θ_s _*and a prior on *ρ_s _*[26]: $\pi \left(\theta_{s}\right)=\frac{\theta_{s}^{\alpha_{\theta_{s}}-1}{\left(1-\theta_{\text{s}}\right)}^{\beta_{\theta_{s}}-1}}{B\left(\alpha_{\theta_{s}},\beta_{\theta_{s}}\right)}$ and $\pi \left(\rho_{s}\right)=\frac{\rho_{s}^{\alpha_{\rho_{s}}-1}{\left(1-\rho_{s}\right)}^{\beta_{\rho_{s}}-1}}{B\left(\alpha_{\rho_{s}},\beta_{\rho_{s}}\right)}$, where *α*(·) and *β*(·) are hyper-parameters in the prior distributions [26-28]. Then, the marginal distribution of $c_{s}^{+}$ is

$$m\left(c_{s}^{+}\right)=C_{\frac{N}{2}}^{c_{s}^{+}}\frac{\Gamma \left(\alpha_{\theta_{s}},\beta_{\theta_{s}}\right)}{\Gamma \left(\alpha_{\theta_{s}}\right)\Gamma \left(\beta_{\theta_{s}}\right)}\frac{\Gamma \left(\alpha_{\theta_{s}}+c_{s}^{+}\right)\Gamma \left(\frac{N}{2}-c_{s}^{+}+\beta_{\theta_{s}}\right)}{\Gamma \left(\alpha_{\theta_{s}}+\beta_{\theta_{s}}+\frac{N}{2}\right)}$$

The marginal distribution of $c_{s}^{-}$ is similar. The probability of the observed genotypes on *s *is equal to the sum of $\frac{m\left(c_{s}^{\cdot }\right)}{C_{\frac{N}{2}}^{c_{s}^{\cdot }}}$ Thus we have:

$$P\left(Y_{s}|X_{s}=1\right)=\frac{\Gamma \left(\alpha_{\theta_{s}},\beta_{\theta_{s}}\right)}{\Gamma \left(\alpha_{\theta_{s}}\right)\Gamma \left(\beta_{\theta_{s}}\right)}\frac{\Gamma \left(\alpha_{\theta_{s}}+c_{s}^{+}\right)\Gamma \left(\frac{N}{2}-c_{s}^{+}+\beta_{\theta_{s}}\right)}{\Gamma \left(\alpha_{\theta_{s}}+\beta_{\theta_{s}}+\frac{N}{2}\right)}\times \frac{\Gamma \left(\alpha_{\rho_{s}},\beta_{\rho_{s}}\right)}{\Gamma \left(\alpha_{\rho_{s}}\right)\Gamma \left(\beta_{\rho_{s}}\right)}\frac{\Gamma \left(\alpha_{\rho_{s}}+c_{s}^{-}\right)\Gamma \left(\frac{N}{2}-c_{s}^{-}+\beta_{\rho_{s}}\right)}{\Gamma \left(\alpha_{\rho_{s}}+\beta_{\rho_{s}}+\frac{N}{2}\right)}$$

On the other hand, if *X_s _*= 0, then there is no PAR between *θ_s _*and *ρ_s _*that infers *θ_s _*= *ρ_s_*. Here, we simply use *ρ_s _*to denote the MAF of *s *for both the cases and controls. Thus, we have

$$P\left(Y_{s}|X_{s}=0\right)=\frac{\Gamma \left(\alpha_{s},\beta_{s}\right)}{\Gamma \left(\alpha_{s}\right)\Gamma \left(\beta_{s}\right)}\frac{\Gamma \left(\alpha_{s}+c_{s}\right)\Gamma \left(N-c_{s}+\beta_{s}\right)}{\Gamma \left(\alpha_{s}+\beta_{s}+N\right)}$$

where $c_{s}=c_{s}^{+}+c_{s}^{-}$. We have now obtained all the three emission probabilities of this HMRF: *p *(*Y*|*X*), *P *(*Y_s_*|*X_s _*= 0) and *P *(*Y_s_*|*X_s _*= 1).

### Estimation of the transition probabilities in HMRF

The transition probabilities link the hidden states *X *and *R*. Let $c_{X}^{+}$ be the counts of the causal variants on all of the elevated regions, and let *c_E _*be the number of variants in those regions. Let $c_{X}^{-}$ be the counts of the causal variants on all of the background regions, and *c_B _*be the number of variants in those regions. Then, we draw two binomial distributions: $c_{X}^{+}\sim \text{Bin}\left(c_{E},\;\xi \right);c_{X}^{-}\sim \text{Bin}\left(c_{B},\;\zeta \right)$ where *ξ *= *P *(*X *= 1|*R *= 1) and *ζ *= *P *(*X *= 1|*R *= 0). We also place the prior distributions on *ξ *and *ζ*, as follows:

$$c_{X}^{+}\sim \text{Bin}\left(c_{E},\;\xi \right);f\left(c_{X}^{+}|\xi \right)=C_{c_{E}}^{c_{X}^{+}}\xi^{c_{X}^{+}}{\left(1-\xi \right)}^{c_{E}-c_{X}^{+}}$$

and

$$c_{X}^{-}\sim \text{Bin}\left(c_{B},\;\zeta \right);f\left(c_{X}^{-}|\zeta \right)=C_{c_{B}}^{c_{X}^{-}}\zeta^{c_{X}^{-}}{\left(1-\zeta \right)}^{c_{B}-c_{X}^{-}}$$

where *ξ *= *P *(*X *= 1|*R *= 1) and *ζ *= *P *(*X *= 1|*R *= 0). We also place the prior distributions on *ξ *and *ζ*, as follows:

$$\pi \left(\xi \right)=\frac{\xi^{\alpha_{\xi }-1}{\left(1-\xi \right)}^{\beta_{\xi }-1}}{B\left(\alpha_{\xi },\beta_{\xi }\right)};\pi \left(\zeta \right)=\frac{\zeta^{\alpha_{\zeta }-1}{\left(1-\zeta \right)}^{\beta_{\zeta }-1}}{B\left(\alpha_{\zeta },\beta_{\zeta }\right)}$$

where *α*(·) and *β*(·) are also hyper-parameters.

Thus, we have the conditional probability of *X *given *R*:

$$P\left(X|R\right)=\frac{\Gamma \left(\alpha_{\xi },\beta_{\xi }\right)}{\Gamma \left(\alpha_{\xi }\right)\Gamma \left(\beta_{\xi }\right)}\frac{\Gamma \left(\alpha_{\xi }+c_{X}^{+}\right)\Gamma \left(c_{E}-c_{X}^{+}+\beta_{\xi }\right)}{\Gamma \left(\alpha_{\xi }+\beta_{\xi }+c_{E}\right)}\times \frac{\Gamma \left(\alpha_{\zeta },\beta_{\zeta }\right)}{\Gamma \left(\alpha_{\zeta }\right)\Gamma \left(\beta_{\zeta }\right)}\frac{\Gamma \left(\alpha_{\zeta }+c_{X}^{-}\right)\Gamma \left(c_{B}-c_{X}^{-}+\beta_{\zeta }\right)}{\Gamma \left(\alpha_{\zeta }+\beta_{\zeta }+c_{B}\right)}$$

and the posterior distribution of ξ given $c_{X}^{+}$ is

$$\pi \left(\xi |c_{X}^{+}\right)=\frac{\xi^{\alpha_{\xi }+c_{X}^{+}-1}{\left(1-\xi \right)}^{\beta_{\xi }+M-c_{X}^{+}-1}}{B\left(\alpha_{\xi }+c_{X}^{+},\beta_{\xi }+M-c_{X}^{+}\right)}$$

Similarly, the posterior distribution of ζ given $c_{X}^{-}$ is

$$\pi \left(\zeta |c_{X}^{-}\right)=\frac{\zeta^{\alpha_{\zeta }+c_{X}^{-}-1}{\left(1-\zeta \right)}^{\beta_{\zeta }+M-c_{X}^{-}-1}}{B\left(\alpha_{\zeta }+c_{X}^{-},\beta_{\zeta }+M-c_{X}^{-}\right)}$$

Thus far, we have obtained all of the three transition probabilities of this HMRF: *p *(*X*|*R*), $\pi \left(\xi |c_{X}^{+}\right)$ and $\pi \left(\zeta |c_{X}^{-}\right)$.

### Estimation the model parameters

Based on the Gibbs-Markov Equivalence [21], a pseudo-likelihood estimation cycle can be applied to this hidden MRF to estimate the model parameters and update the hidden states. We use the pseudo-likelihood estimation because *p *(*X*; Φ) and *p *(*R*; Φ*_R_*) are difficult to compute directly. The algorithm involves the following four steps:

• Step 1: Estimate *α_θ _*and *β_ρ _*with $\hat{\theta }$ and $\hat{\rho }$ by maximizing the likelihood $L\left(Y/\hat{X}\right)$. Update ${\hat{\theta }}_{s}$ by maximizing the posterior distribution:

$$\pi \left(\theta_{s}|c_{s}^{+}\right)=\frac{\theta_{s}^{\alpha_{\theta_{s}}+c_{s}^{+}-1}{\left(1-\theta_{s}\right)}^{\beta_{\theta_{s}}+N-c_{s}^{+}-1}}{B\left(\alpha_{\theta_{s}}+c_{s}^{+},\beta_{\theta_{s}}+N-c_{s}^{+}\right)}$$

Similarly, Update ${\hat{\rho }}_{s}$.

• Step 2: Estimate *α_ξ_, β_ξ _*and *α_ζ_, β_ζ _*with $\hat{\xi }$ and $\hat{\zeta }$ by maximizing the transition probability $L\left(X/\hat{R}\right)$. Update $\hat{\xi }$ and $\hat{\zeta }$ by maximizing the transition probabilities $\pi \left(\xi |c_{X}^{+}\right)$ and $\pi \left(\xi |c_{X}^{+}\right)$, respectively.

• Step 3: Estimate Φ and Φ*_R _*with $\hat{\Phi }$ and ${\hat{\Phi }}_{R}$ by maximizing the pseudo-likelihood functions:

$$L\left(\hat{X};\Phi \right)=exp\left(\overset{M}{\underset{S}{\sum }}p_{s}\left({\hat{X}}_{s}|{\hat{X}}_{n\left(s\right)}\right);\Phi \right)$$

and $L\left(\hat{R};\Phi_{R}\right)$.

• Step 4: Update $\hat{X}$ and $\hat{R}$ by

$$P\left(X_{s}|Y,{\hat{X}}_{S/s}\right)\propto f\left(Y_{s}|X_{s};\hat{\theta },\hat{\rho }\right)p_{s}\left(X_{s}|{\hat{X}}_{n\left(s\right)};\hat{\Phi }\right)$$

and $P\left(R_{s}|X,{\hat{R}}_{S/s}\right)$.

There are several ways to exit from this iteration. We measure the Euclidean distance between the current and the updated $\hat{X}$. If the distance is less than a preset threshold, our approach will stop the iteration. After the convergence of HMRF, we obtain the estimations of *X *and *R*, as well as the MAFs for every variant. The collapsed rare variants can be tested based on the existing statistics, e.g. in [9,10,14].

### Experiments and results

In this section, we apply our approach on a real dataset from [30] and also compare it with three other approaches using different types of simulated datasets. The three comparison approaches are *RareCover*, which is based on [19], *RWAS *from [14] and *LRT *from [9]. Additionally, it seems that *RareCover *is not released online, so as in many previous works, we re-implement this algorithm and the related statistics by ourselves.

### Simulation frameworks

As the simulation settings in different papers [9,14,19] are quite different, we adopt all of them and generate three types of simulated datasets. In the first one, each dataset has a fixed number of causal variants, while in the second dataset, the number of causal variants is determined by allelic population attributable risk (PAR). The last simulation method first generates elevated regions and background regions and then plants causal variants in each region. We describe the three simulation methods in the following sections.

#### Fix number of causal variants

First, we generate the datasets with fixed numbers of causal variants, following previous approaches [14] and [9]. Each variant is generated independently because they believe that rare variants do not show significant linkage disequilibrium [9,14]. For each variant, the probability distribution of the MAF of site *s *on controls, *ρ_s_*, satisfies the Wright's distribution under purifying selection [4],

$$f\left(\rho_{s}\right)\propto {\left(\rho_{s}\right)}^{\beta_{s}-1}{\left(1-\rho_{s}\right)}^{\beta_{N}-1}e^{\sigma -\rho_{s}\sigma }$$

where *σ *is the selection coefficient, *β_S _*is the probability that the normal allelic site mutates to the causal variant, and *β_N _*is the probability that a causal variant repairs to a normal variant. We take *σ *= 12.0, *β_S _*= 0.001 and *β_N _*= 0.00033, which are the same settings used by [9,11,14]. Then, the relative risk of *s *is: $RR=\frac{\delta }{\left(1-\delta \right)\rho_{s}}+1$, where *δ *is the marginal PAR. The marginal PAR is equal to the group PAR (Δ) divided by the number of causal variants, while the relative risk of *M *variants is 1 [14]. Afterwards, the MAF of *s *for the cases is calculated according to $\theta_{s}=\frac{RR\times \rho_{s}}{\left(RR-1\right)\rho_{s}+1}$. In each dataset, we simulate *N *= 2000 genotypes with half cases and half controls. The mutations on the cases and the controls are sampled independently according to *θ_s _*and *ρ_s_*, respectively.

#### Causal variants depends on PAR

The second way generates a set, *C*, that contains all of the causal variants. Instead of a fixed number, the total number of causal variants depends on PAR [19], which is limited by Δ (the group PAR):

$$\Delta \geq 1-\underset{s\in C}{\prod }\left(1-\frac{\theta_{s}Pr}{P_{D}}\right)$$

where *Pr *represents the penetrance of the group of causal variants and *P_D _*is the disease prevalence in the population. Different settings are applied in the experiments.

We use the algorithm proposed in [19] to obtain the MAF of each causal variant. The algorithm samples the MAF of a causal variant *s, θ_s_*, from the Wright's distribution with *σ *= 30.0, *β_S _*= 0.2 and *β_N _*= 0.002 [4,19], and then appends *s *to *C*. Next, the algorithm checks whether $\prod_{s\in C}\left(1-\frac{\theta_{\text{s}}Pr}{P_{D}}\right)>1-\Delta$ is true. If the inequality does not hold, the algorithm terminates and outputs *C*. Thus, we obtain all of the causal variants and their MAFs. If the inequality holds, then the algorithm continuously samples the MAF of the next causal variant. The mutations on genotypes are sampled according to *θ_s_*.

For those non-causal variants, we use Fu's model [29] of allelic distributions on a coalescent, which is the same used in [19]. We adopt $\rho_{s}=\frac{5.0}{N}$. The mutations on genotypes are sampled according to *ρ_s_*. The phenotype of each individual (genotype) is computed by the penetrance of the subset, *Pr*. Thereafter, we sample 1000 of the cases and 1000 of the controls.

#### Causal variants depends on regions

There are many ways to generate a dataset with regions. The simplest way is to preset the elevated regions and the background regions and to plant causal variants based on certain probabilities. An alternate way creates the regions by a Markov chain. For each site, there are two groups of states. The *E state *denotes that the variant is located in an *elevated *region, while the *B state *denotes that the variant is located in a *background *region. Both states *E *and *B *can transfer to a causal state *C *or a non-causal state $\bar{C}$. If the Markov chain travels to the $\bar{C}$ state, it plants a mutant on the genotype with probability *ρ*. If the variant is considered to be causal, it may continuously transfer to the state *A*, which means that the genotype carries a mutant that may affect the phenotype with penetrance *Pr*. Otherwise it arrives in the state *Ā*, and the Markov chain plants a mutant or a wild-type allele on the genotype afterwards.

To generate enough genotypes, we perform the following steps for each variant: if the process drops into $\bar{C}$, we take 50,000 iterations to yield a mutant, where *ρ *is sampled from the Wright's distribution with *σ *= 30.0, *β_S _*= 0.2 and *β_N _*= 0.002. If it drops into *A *or *Ā*, we design an iteration to *C *until it reaches 50,000 iterations. The transition probability from *C *to *A *is equal to *ρ × Pr*. After we have enough genotypes, we sample 1000 cases and 1000 controls from them.

### Comparisons on power

Similar to the measurements in [9,14], the power of an approach is measured by the number of significant datasets, among many datasets, using a significance threshold of 2.5 × 10^-6 ^based on the Bonferroni correction assuming 20000 genes, genome-wide. We test at most 1000 datasets for each comparison experiment.

#### Power versus different proportions of causal variants

We compare the powers under different sizes of total variants. In the first group of experiments, we include 50 causal variants and vary the total number of variants from 100 to 5000. Thus, the proportions of causal variants decrease from 50% to 1%. In the second group of experiments, we hold the group PAR as 5% and vary the total number of variants as before. The results are compared in Table 1. From the results, our approach clearly shows more powerful and more robust at dealing with large-scale data. We also test our approach on different settings of the group PARs. Those results can be found in Table S1 in the Additional file 1.

**Table 1:** The power comparisons at different proportions of causal variants

Total	Causal	RareProb	RareCover	RWAS	LRT
100	50	100%	100%	100%	100%
200	50	100%	100%	99.6%	99.9%
400	50	100%	100%	85.3%	88.6%
600	50	100%	94.6%	54.1%	58.8%
800	50	100%	0.0%	33.0%	36.5%
1000	50	100%	0.0%	20.7%	22.0%
2000	50	100%	0.0%	2.0%	2.0%
3000	50	100%	0.0%	0.8%	0.0%
4000	50	100%	0.0%	0.4%	0.0%
5000	50	100%	0.0%	0.3%	0.0%

200	1*	51.0%	0.0%	0.0%	0.0%
400	3*	77.0%	0.0%	0.0%	0.0%
600	2*	63.6%	0.0%	0.0%	0.0%
800	3*	57.1%	0.0%	0.0%	0.0%
1000	3*	59.0%	0.0%	0.0%	0.0%
2000	1*	34.0%	0.0%	0.0%	0.0%
3000	2*	41.2%	0.0%	0.0%	0.0%
4000	3*	40.0%	0.0%	0.0%	0.0%
5000	2*	29.8%	0.0%	0.0%	0.0%

The upper section of this table shows the results with a fixed number of causal variants. The column "Causal" shows the number of causal variants, and "*" indicates that the value is an average value."

The Type I error rate is another important measurement for estimating an approach. To compute the Type I error rate, we apply the same technique as [19]. Type I error rate is defined as the probability of a non-causal variant being selected in the potential causal set. We compare our approach only with *RareCover *because *RWAS *or *LRT *does not select any potential causal variants. The results on different configurations can be found in supplementary documents. Based on the results, our approach always holds reasonable Type I error rates. Although on some configurations *RareProb *has a little higher Type I error rates, e.g. 1%-10% higher when gourp PAR is 5%, than *RareCover*, the absolute values are still satisfied. Moreover, when the group PAR decreases, *RareProb *always performs lower Type I error rates than *RareCover*. These results can be found in Table S2 in the Additional file 2. Considering both statistical power and Type I error rate, the advantage of *RareProb *cannot be neglected: it is able to identify most of the causal variants with an acceptable Type I error rate. In the other words, if an approach rarely identifies correct variants, a low Type I error rate becomes meaningless.

#### Power versus different configurations of regions

We compare the powers on different configurations of elevated regions and background regions and test the performance of our approach in identifying the regions. At each total variant number, we preset the number of regions between 2 and 8, with half elevated regions and half background regions. In these datasets, the probability of a rare variant being causal is 0.1 if the variant is located in an elevated region; otherwise, the probability is 0.001 if variant is located in a background region. In the last group of experiments, the regions are generated by the Markov chain, where the transition probability of remaining in the same regions (keeps in elevated region or background region) is 0.8, while the transition probability of transitioning between different regions (jumps from an elevated region to a background region, or jumps from a background region to an elevated region) is 0.2. The emission probabilities are the same as before. We test the powers and record the percentages of correct identifications on the regions. The results are listed in Table 2. The results show that our approach successfully estimates the regions, while *RareCover *suffers difficulty on identifying neither candidate causal variants nor region information. We also test our approach on total variants being 3000, 4000 and 5000. These results can be found in Table S3 in the Additional file 3.

**Table 2:** The power comparisons for different configurations of regions

Total	Causal	Regions	Length	RareProb	Correct R
1000	36*	1	50	100%	96%
	37*	2	50	100%	98%
	36*	3	50	100%	97%
	35*	4	50	100%	98%

2000	73*	1	100	100%	97%
	73*	2	100	100%	97%
	70*	3	100	100%	98%
	71*	4	100	100%	96%

Total	Causal	Regions	Length	RareCover	Correct R

1000	36*	1	50	0.0%	1.9%
	37*	2	50	0.0%	1.4%
	36*	3	50	0.0%	1.7%
	35*	4	50	0.0%	1.6%

2000	73*	1	100	0.0%	0.7%
	73*	2	100	0.0%	0.8%
	70*	3	100	0.0%	1.3%
	71*	4	100	0.0%	0.8%

The column "Causal" represents the total number of causal variants, "Region" denotes the total number of elevated regions, "Length" indicates the total number of variants locating in elevated regions. The column "Correct R" shows the percentage of correct identification of regions.

### RareProb on real mutation screening data

Finally, we apply our approach to a real mutation screening dataset. This dataset has been previously published by [30]. Authors screen for a susceptibility gene, *ATM*, which is thought to associate with *ataxia telangiectasia*. *ATM *is also an intermediate-risk susceptibility gene for breast cancer [9,14]. The dataset (ATM_CCMSdata_Dec2011_v1) we have consists of 121 rare variants in a set of 2506 cases and 2235 controls, which is called "bona fide case-control studies" [9,14].

We apply *RareProb *to this dataset without any prior information. *RareProb *identifies variant #c.4424A *>*G as a causal variant and reports a significant association with a *p*-value of 8.8817 × 10^-16^. As a comparison, authors in [30] reports that they did identify a significant association with the help of the prior information, but that they did not find a significant association only according to the results of *CMC*. Sul and others [14] applied *RWAS *and reports a non-significant association with *p*-value of 0.3946 without prior information and a non-significant association with *p*-value of 0.0078 and 0.0881 when prior information of variants is obtained by *Align-GVGD *[22] and *SIFT *[23], respectively. Sul and others [9] also applied *LRT *and reports that a non-significant association with *p*-value of 0.3934 was found without prior information, but a significant association with *p*-value of 0.0058 and 0.08384 were found introducing *Align-GVGD *scores and *SIFT *scores, respectively. Our approach successfully identifies an association and clearly points out the candidate causal variant, without prior information, while either *RWAS *or *LRT *cannot achieve this.

## Conclusion

In this article, we propose a probabilistic method, *RareProb*, to identify multiple rare variants that contribute to dichotomous disease susceptibility. Our approach is inspired by *RareCover*. Both approaches select a subset of potentially causal variants from the given variants, which means our approach does not rely on the pre-selection of candidate rare variants. Furthermore, as opposed to simply merging the variants in *RareCover*, our approach gains power by considering the directions and the magnitudes of the genetic effects. Both the causal and the protective variants can be described by pair-wise measurements, respectively. This method gets rid of the weakness of losing statistical power when "causal", "neutral" and "protective" variants are combined. Note that the pair-wise weight is not the linkage disequilibrium (LD). LD is quite difficult to observe, although it is expected among rare variants. The pair-wise measurements indicate the likelihood of two variants being collapsed, which is similar to the kernel functions in regression-based frameworks. This weight is then used to build up the neighborhood system of the hidden Markov random field model.

The Markov random field model treats all of the variants as one vector and estimates their causal/non-causal status by globally maximizing the likelihood of genotypes instead of by local optimization. Our approach gains more power than existing group-wise collapsing approaches; *RareProb *filters out those variants with non-causal status. At the same time, unlike the previous selection-based approaches, *RareProb *controls the false positive rate by partitioning elevated regions and background regions, instead of by presetting any sliding windows. Regions are much more flexible than preset sliding windows. While existing approaches can only handle hundreds of variants, there is no doubt that the total number of variants will increase rapidly with the development of new technologies, e.g. applications of next generation sequencing. The simulation experiments show that our approach obtains significantly more power, especially when the total number of given rare variants is large. We also apply our approach to a real mutation screening dataset and a significant association is found. Our approach is able to handle thousands of variants. Moreover, our approach is easy to extend to an "additive" genetic model and multiple phenotypes by updating the Dirichlet prior distribution.

## Competing interests

The authors declare that they have no competing interests.

## Authors' contributions

JW and ZM conducted this research. JW designed algorithms and experiments. ZC, AY and JZ developed the software packages and participated in the performance analysis and the experiments on the real dataset. JW, ZM and JZ wrote this paper. All authors have read and approved the final manuscript.

## Declarations

The publication costs for this article were funded by Xi'an Jiaotong University.

This article has been published as part of *BMC Genomics *Volume 14 Supplement 1, 2013: Selected articles from the Eleventh Asia Pacific Bioinformatics Conference (APBC 2013): Genomics. The full contents of the supplement are available online at http://www.biomedcentral.com/bmcgenomics/supplements/14/S1.

## Supplementary Material

Additional file 1 **Table S1**. The power comparisons at different levels of PAR and different numbers of causal variants. Click here for file

Additional file 2 **Table S2**. The power comparisons for different configurations of causal variants depended on PARs. Click here for file

Additional file 3 **Table S3**. The power comparisons for different configurations of regions. Click here for file
